# Reversine and herbal Xiang–Sha–Liu–Jun–Zi decoction ameliorate thioacetamide-induced hepatic injury by regulating the RelA/NF-κB/caspase signaling pathway

**DOI:** 10.1515/biol-2020-0059

**Published:** 2020-09-15

**Authors:** Zhen-Hao Mai, Yu Huang, Di Huang, Zi-Sheng Huang, Zhi-Xiang He, Pei-Lin Li, Shuai Zhang, Jie-Feng Weng, Wei-Li Gu

**Affiliations:** Department of Surgery, Guangzhou First People's Hospital, School of Medicine, South China University of Technology, No.1 Panfu Road, Yuexiu District, Guangzhou, Guangdong 518180, People’s Republic of China; Guangzhou Medical University, Guangzhou, Guangdong 510180, People’s Republic of China; Guangzhou Digestive Disease Center, Guangzhou First People’s Hospital, Guangzhou, Guangdong 510180, People’s Republic of China

**Keywords:** liver fibrosis, liver index, reversine, NF-κB, caspase 1, interleukins, Xiang–Sha–Liu–Jun–Zi decoction

## Abstract

This study investigated the anti-fibrotic effects of reversine and Chinese medicine Xiang–Sha–Liu–Jun–Zi decoction (XSLJZD) on thioacetamide (TAA)-induced hepatic injury. Sprague-Dawley rats were intraperitoneally administered with TAA, then injected with reversine intraperitoneally, and/or orally provided with XSLJZD. TAA resulted in liver injury with increases in the liver index and levels of serum aspartate aminotransferase (AST) and alanine aminotransferase. Reversine alleviated the liver index and AST level and improved TAA-induced pathological changes but decreased TAA-induced collagen deposition, and α-smooth muscle actin and transforming growth factor-β1 expression. Reversine also modulated the mRNA levels of inflammatory cytokines, such as RelA, interleukin (IL)-17A, IL-22, IL-1β, IL-6, NLR family pyrin domain containing 3, platelet-derived growth factor, and monocyte chemoattractant protein, and suppressed nuclear factor (NF)-κB (p65) phosphorylation and caspase 1 activation. Meanwhile, XSLJZD protected TAA-injured liver without increasing fibrosis and enhanced the regulating effect of reversine on RelA, IL-17A, IL-1β, and MCP-1 cytokines. In conclusion, reversine ameliorates liver injury and inhibits inflammation reaction by regulating NF-κB, and XSLJZD protects the liver through its synergistic effect with reversine on regulating inflammatory cytokines.

## 1 Introduction

Liver fibrosis is an over self-repairing pathophysiological response to acute or chronic liver damage caused by infection, drug toxicity, metabolic disorders, or immune reactions [1,2]. Hepatic stellate cells (HSCs) are suggested as the primary effectors of extracellular matrix (ECM) deposition in normal and fibrotic livers [3] and activate the immune response by secreting cytokines and chemokines and by interacting with immune cells. The inflammatory response generally occurs during the development of liver fibrosis, and inflammatory cytokines can promote liver fibrosis progression [4,5,6]. Liver inflammation is one of the driving factors of liver fibrosis [7].

Many inflammatory factors are closely related to liver fibrosis. Nuclear factor-κB (NF-κB), a transcription factor composed of homo- or heterodimers of the Rel protein family, can be persistently stimulated following HSC activation [8]. As a result, NF-κB responsive genes such as interleukin (IL)-6 and intercellular adhesion molecule-1 are constitutively expressed in activated HSCs. In addition, NF-κB is a major downstream effector of tumor necrosis factor-α (TNF-α). Its upregulation by TNF-α can modulate the hepatic oxidative stress cascade to some extent and consequently activate target genes, including chemokines, cytokines, multi-peptides, or proteins. In HSCs, TNF-α activates the transcription factor AP-1 and c-Jun N-terminal kinase (JNK), increases matrix metalloproteinase gene expression, and reduces collagen gene expression [8]. Transforming growth factor (TGF)-β activates Ras and Raf-1, followed by extracellular signal-regulated kinase activation in HSCs through a platelet-derived growth factor (PDGF)-independent mechanism [8]. TGF-β can also activate p38 mitogen-activated protein kinase (MAPK) signaling, which is mediated by the upstream TGF-β-activated kinase-1 and MKK3/6, partially facilitating the TGF-β-induced type I collagen gene expression in HSCs [9,10,11]. ILs, another series of key factors, can initiate and accelerate liver fibrosis by stimulating liver inflammatory response. During fibrogenesis, IL-1 mainly activates HSCs and Kupffer cells through autocrine or paracrine regulation, thereby promoting the HSC expression of matrix metallopeptidase (MMP) 13 and the tissue inhibitor of metalloproteinase-1, which regulates the balance of the ECM. IL-1 can also promote HSC proliferation by increasing the formation of the cell cycle S phase. Meanwhile, IL-17 also has a strong pro-inflammatory property and a possible synergistic effect with other cytokines such as TNF-α and IL-1 to stimulate the formation of the ECM. Our previous study also showed that blocking IL-17A by monoclonal antibodies substantially improves liver function and decreases hepatocellular necrosis, pro-inflammatory cytokines, neutrophils and macrophage influx in a bile duct ligation-induced liver injury and fibrosis mouse model [12].

Liver fibrosis is believed to be reversible [13] and small chemical compounds that offer a promising choice are currently being tested in early-phase clinical trials [14]. The small molecule reversine, a synthetic 2,6-disubstituted purine analog, attracted our attention because of its potential to induce the differentiation and inhibit the proliferation of multiple cells [15]. Reversine inhibits MMP-3, IL-6, and IL-8 expression by suppressing reactive oxygen species (ROS) and JNK/AP-1 activation in IL-1β-stimulated human gingival fibroblasts [16]. Lee et al. [17] reported that reversine inhibits MMP-1 and MMP-3 expressions by suppressing ROS/MAPK/AP-1 activation in UV-stimulated human keratinocytes and dermal fibroblasts. Recent studies also showed that reversine affects the caspase-dependent apoptosis signaling pathway [18]. As suggested by its name, we aimed to determine whether reversine could reverse liver fibrosis. In our previous *in vitro* study, we found that this molecule inhibits the proliferation of HSCs, promotes their apoptosis, and affects the expression of TGF-β1 and α-smooth muscle actin (α-SMA), indicating its potential anti-fibrosis role [19].

Traditional Chinese medicines (TCMs) protect the liver due to their anti-inflammatory and anti-oxidative effects [20]. Xiang–Sha–Liu–Jun–Zi decoction (XSLJZD) can eliminate the edema of the gastric mucosa and reduce inflammatory cell infiltration [21]. At present, this TCM is mostly used to treat various inflammatory diseases, especially in the digestive system [22,23,24]. Chinese research on XSLJZD for the treatment of liver disease also showed that it can remarkably reduce blood lipids and has a good clinical effect to cure non-alcoholic fatty liver. XSLJZD also has a certain effect on cirrhosis and ascites and a specific protective activity for liver function.

In this study, the effects of reversine on liver function and fibrosis and the underlying mechanisms in inflammatory signaling pathways were investigated. Results showed that reversine can modulate inflammatory cytokines such as RelA, IL-17A, IL-22, IL-1β, IL-6, NLR family pyrin domain containing 3 (NLPR3), PDGF, and monocyte chemoattractant protein (MCP)-1. Based on its role in reducing inflammatory cell infiltration and regulating cellular and humoral immunity, we hypothesized that XSLJZD has a certain regulatory effect on liver fibrosis inflammation. We tested the combined effects of reversine and XSLJZD and found their synergistic influence on thioacetamide (TAA)-induced rat liver injury.

## 2 Materials and methods

### 2.1 Animals

Male Sprague-Dawley rats (8 weeks old, weighing 220–250 g) were purchased from the Experimental Animal Center of Sun Yet-sen University, China.

### 2.2 Reagents

Reversine (2-(4-morpholinoanilino)-6-cyclohexylaminopurine, CAS no. 656820-32-5) was purchased from Cayman Company and dissolved in dimethyl sulfoxide (Sigma-Aldrich, USA) following the instructions. TAA was purchased from Tianjin Damao Chemical Reagent Factory. *Radix Ginseng* (*Panax ginseng* C. A. Mey., Araliaceae), *Rhizoma Atractylodis macrocephalae* (*Atractylodes macrocephala* Koidz., Asteraceae), *Poria* (*Poria cocos* (Schw.) Wolf., Polyporaceae), *Radix Glycyrrhizae* (*Glycyrrhiza uralensis* Fisch., Fabaceae), *Pericarpium Citri reticulatae* (*Citrus reticulata* Blanco, Rutaceae), *Rhizoma Pinelliae* (*Pinellia ternata* (Thunb.) Breit., Araceae), *Fructus Amomi* (*Amomum villosum* Lour., Zingiberaceae), *Radix Aucklandiae* (*Aucklandia lappa* Decne., Asteraceae), and *Rhizoma Zingiberis recens* (*Zingiber officinale* Rosc., Zingiberaceae) herbs were acquired from Guangzhou First People’s Hospital and prepared according to the Herbal Pharmacopoeia and Pharmacopoeia of the People’s Republic of China.

### 2.3 XSLJZD preparation

In brief, 3 g of *Radix Ginseng*, 6 g of *Rhizoma Atractylodis macrocephalae*, 6 g of *Poria*, 2 g of *Radix Glycyrrhizae*, 2.5 g of *Pericarpium Citri reticulatae*, 3 g of *Rhizoma Pinelliae*, 2.5 g of *Fructus Amomi*, 2 g of *Radix Aucklandiae*, and 6 g of *Rhizoma Zingiberis recens* were weighed and washed once. The herbs were marinated in cool water at room temperature for half an hour, collected in a multifunctional electronic boiling pot with 500 mL of water, and decocted up to 100°C for 10 min. The decoction liquid was collected in 50 mL tubes after being filtered using four layers of ordinary cotton. The XSLJZD was then stored at 4°C before use [23].

### 2.4 Establishment of the liver injury model and groups

The liver injury model was established by intraperitoneally (i.p.) injecting 200 mg/kg TAA solution in rats twice per week for 4 weeks. The TAA-injected rats were randomly divided into the TAA group, low reversine group (i.p. 50 µg/kg body weight, RE-l), and high reversine group (i.p. 200 µg/kg body weight, RE-h). Then reversine was intraperitoneally injected into rats. The normal rats with neither TAA nor reversine treatment were used as the control (CON) group. Five rats in each group were checked. For the decoction group (XSLJZD), five TAA-injected rats were orally administered with 10 ml/kg XSLJZD. For the RE-h + XSLJZD group, another five TAA-injected rats were injected with 200 µg/kg reversine solution and simultaneously provided with 10 ml/kg XSLJZD for the same periods. Each group was treated three times per week for 3 weeks.


**Ethical approval:** The research related to animal use has been complied with all the relevant national regulations and institutional policies for the care and use of animals and has been approved by the Animal Research Ethics Committee in Guangzhou First People’s Hospital.

### 2.5 Liver index

Body weight was measured under anesthesia before mouse sacrifice. The liver was extracted and weighed, and the liver index was calculated according to the following equation: liver index (%) = liver weight (g)/body weight (g) × 100.

### 2.6 Biochemical assay of blood samples

Blood was collected from the inferior vena cava at the time of sacrifice, and serum was obtained through centrifugation (3,000 rpm for 5 min). Alanine aminotransferase (ALT) and aspartate aminotransferase (AST) levels were detected in the clinical laboratory of Guangzhou First People’s Hospital.

### 2.7 Hematoxylin and eosin (H&E) staining

Liver tissues were fixed with 4% paraformaldehyde (Wako Pure Chemical Industries Ltd, Osaka, Japan) for 1–3 days, dehydrated with high concentrations of ethanol, embedded in paraffin (Paraplast Plus; Leica Biosystems, Inc., Richmond, IL, USA), and cut into sections of 4 µm thickness. After deparaffinization, the specimens were stained for 3 min in hematoxylin (Sigma-Aldrich, Munich, Germany) and washed twice with H_2_O, followed by incubation with 1% acidic alcohol for 3 s and re-staining with running tap water for 10 min. Then, the cytoplasm of specimens was stained with eosin (Sigma-Aldrich, Munich, Germany) for 10 s. The sections were dehydrated twice through incubation in 96% EtOH for 5 min, followed by another incubation in 100% EtOH for 5 min twice. Thereafter, the specimens were incubated in Roti^®^-Histol for 10 min, and the sections were mounted using a water-free mounting medium (Entellan^®^).

### 2.8 Collagen fiber detection

The collagen fibers in liver tissues were detected in the sections stained with Masson’s trichrome kit (Cosmobio, Japan) following the standard procedure. The collagen fiber area was stained blue. Histological quantification of the collagen areas was calculated from images captured through a 4× lens by using an optical microscope (BX53; Olympus, Tokyo). At least five images for each rat were acquired using the imaging software developed at the National Institutes of Health (ImageJ). The percentage collagen area was calculated as the ratio of the collagen fiber area to the whole image area in each group.

### 2.9 Immunohistochemical staining

The liver tissues were fixed in 10% neutral buffered formalin solution, embedded in paraffin, and stained for routine histology. The sections were dewaxed in xylene and dehydrated in alcohol. Antigen retrieval was achieved by microwaving in citric saline for 5 min. The sections were then treated with 0.3% H_2_O_2_ for 15 min to block endogenous peroxidase activity, further blocked by 5% bovine serum albumin (BSA), and incubated overnight at 4°C with primary rabbit polyclonal antibodies against α-SMA (Ab5694; 1:200) and TGF-β1 (Ab66043; 1:100). After rinsing with phosphate-buffered saline solution, the sections were incubated with biotinylated secondary antibodies for 60 min at room temperature. α-SMA and TGF-β1 expression was visualized using 3,3′-diaminobenzidine tetrahydrochloride staining. The sections were counterstained with Mayer’s hematoxylin for 5 min and dehydrated. α-SMA and TGF-β1 positive areas within the fibrotic region were observed, and images were captured using an optical microscope (BX53; Olympus, Tokyo). Quantitative analysis of the positively stained areas was conducted using ImageJ software. Integrated optical density (IOD) values were measured from the images acquired using 10× lens. At least five images were captured for each rat.

### 2.10 Quantitative reverse transcription-polymerase chain reaction (qRT-PCR)

Total RNA was extracted from rat liver tissues by using TRIzol reagent and transcribed into cDNA according to the reverse transcription kit. Primer design (Table 1) and synthesis were provided by Guangzhou RuiBo Biotechnology Co., Ltd. PCR was performed at 95°C for 10 min, followed by 40 cycles of amplification at 95°C for 15 s, 61°C for 30 s, and 70°C for 30 s by using CFX96 (Bio-Rad, USA). The expression levels were normalized to that of β-actin (ACTB), and the control sample was set as 1.0, and mRNA expression was determined using the comparative cycle time (ΔΔ^Ct^) method. The experiment was repeated at least three times for three independent RNA samples extracted from at least three lobes.

**Table 1:** Primer sequence and product size of real-time PCR analyses

Gene	Gene ID	Primer sequence		Size (bp)
		Forward	Reverse	
IL-1β	24494	CCTCGTGCTGTCTGACCCAT	GTCGTTGCTTGTCTCTCCTTGTA	112
IL-6	24498	TCACAGAGGATACCACCCACA	TTCCAAGATCTCCCTGAGAACA	89
IL-22	500836	CTGCTTCTCGTTGCTCTGTGG	CGATGTATGGCTGCTGGAAGTT	97
IL-17A	301289	GCTACTGAACCTGGAGGCTACA	CTCGGCGTTTGGACACACT	73
TNF-a	24835	CGATGCTCAGAAACACACGAGA	CCGAAAGCCCATTGGAATC	93
PDGFB	24628	TCGAGCCAAGACACCTCAAACT	CTTGTCATGGGTGTGCTTAAACTT	100
NLRP3	287362	AAGCTCCTCTGTGAGGGACTTC	GCAGCCCTGCTGTTTCAGA	207
MCP-1	24770	CAACCCTAAGGACTTCAGCACC	GGCATCACATTCCAAATCACAC	149
RelA	309165	ACAGACCCAGGAGTGTTCACAG	GTCACCAGGCGAGTTATAGCTT	143

### 2.11 Western blot analysis

Total cellular proteins were extracted from cells in each group by using RIPA lysis buffer (Beyotime, China) and quantified using a bicinchoninic acid (BCA) protein assay kit (Beyotime, China). In brief, 50 protein samples were subjected to standard SDS-PAGE and transferred onto a polyvinylidene difluoride membrane (Millipore, USA). Nonspecific protein binding was blocked by 60 min of incubation in Tris-buffered saline with 0.1% v/v Tween-20 (TBS-T) containing 5% (wt/vol) non-fat milk and 5% BSA. The membranes were then incubated at 4°C overnight with primary antibodies: mouse anti-­NF-κB p65 monoclonal antibody (ABM40053; 1:1,000), rabbit anti-NF-κB p65 phospho Ser536 polyclonal antibody (ABP50373; 1:500), anti-pro caspase 1 + p10 + p12 antibody (ab179515; 1:1,000), and mouse anti-GAPDH antibody (1:5,000) diluted in TBS-T with 5% BSA. After four 10 min washes with TBS-T, the membranes were incubated for 60 min at room temperature with a secondary antibody (ProteinTech, USA). After four additional washes with TBS-T, the protein bands of interest were detected through chemiluminescence using a ClarityTM Western ECL Substrate Kit (BD, USA). The images were acquired using chemiluminescence imaging equipment (GENE GNOME, USA), and scanning densitometry was performed using Image pro plus software (IPP 6.0).

### 2.12 Statistics

All data were expressed as mean ± standard deviation (SD). Statistical analyses were performed with GraphPad Prism 8.0 (GraphPad Software, Inc., CA, USA) using the indicated test presented in detail in figure legends. Differences with *p* values < 0.05 were considered statistically significant.

## 3 Results

### 3.1 Reversine attenuated TAA-induced liver injury

**Figure 1:**
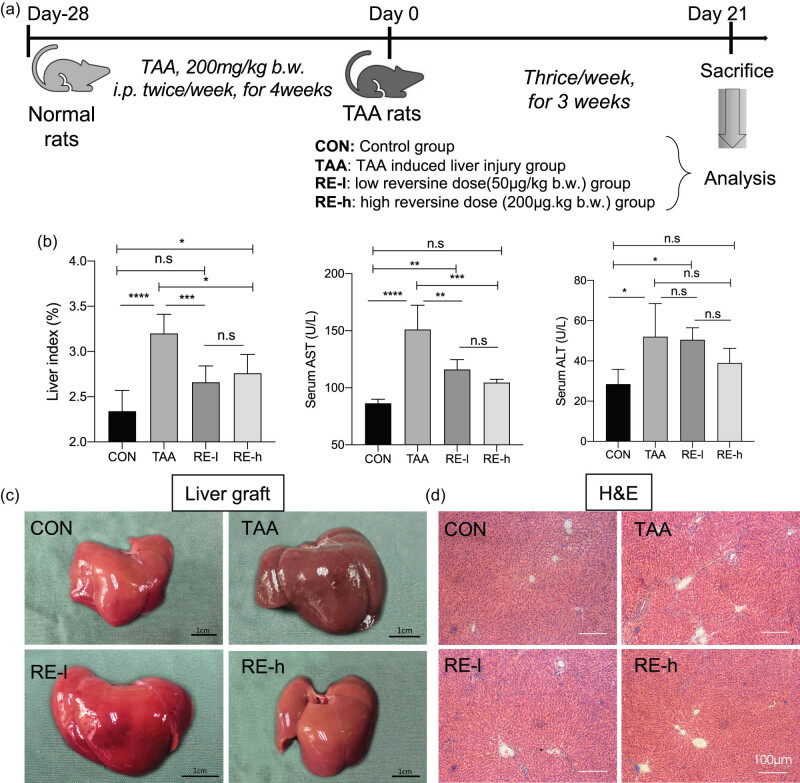
Reversine attenuated TAA-induced liver injury. (a) Schematic representation of the study groups. Reversine at low (50 µg/kg b.w., RE-l) and high density (200 µg/kg b.w., RE-h) was used to treat TAA-injected rats. Normal rats with neither reversine nor TAA treatment were set as CON. (b) Liver index, serum AST level and ALT level are listed in bar graphs. Data are presented as mean ± SD (*n* = 5) and analyzed with the one-way ANOVA test, with *p < 0.05, **p < 0.01, ***p < 0.001, ****p < 0.0001, n.s: no significance. AST: aspartate aminotransferase; ALT: alanine aminotransferase. The general view of the liver graft (c) and the images of H&E staining (d) in each group.

We first investigated the effect of reversine on TAA-induced liver injury (Figure 1a). Compared with the CON group, the TAA-treated group had increased liver index (*p* < 0.0001) and AST (*p* < 0.0001) and ALT (*p* < 0.05) levels (Figure 1b). Reversine treatment decreased the TAA-induced liver index (*p* < 0.05) and AST level (*p* < 0.01) to a comparable normal level but showed no significant influence on the ALT level (*p* > 0.05), which displayed a declining trend (Figure 1b). In the CON group, the liver was reddish-brown and had a smooth appearance without nodules. In the TAA group, the liver was dark brown with a rough surface and sand-like particles (Figure 1c). In the reversine group, the livers were similar to those in the CON group (Figure 1c). Histologically, normal livers were intact, arranged neatly without evidence of degeneration and necrosis in hepatocytes. The structures of hepatic lobules and the portal area were normal and intact in the CON group. Hoverer, the TAA liver had an increase in disordered hepatic lobular structures. Interstitial spaces were found in some areas, and pseudolobule formation can be observed occasionally (Figure 1d). Reversine treatment attenuated the TAA-induced disorder of hepatic lobular structures, which were comparable to the normal livers (Figure 1d). These findings revealed that reversine protected the liver from TAA-induced injury.

### 3.2 Reversine attenuated TAA-induced liver fibrosis

**Figure 2:**
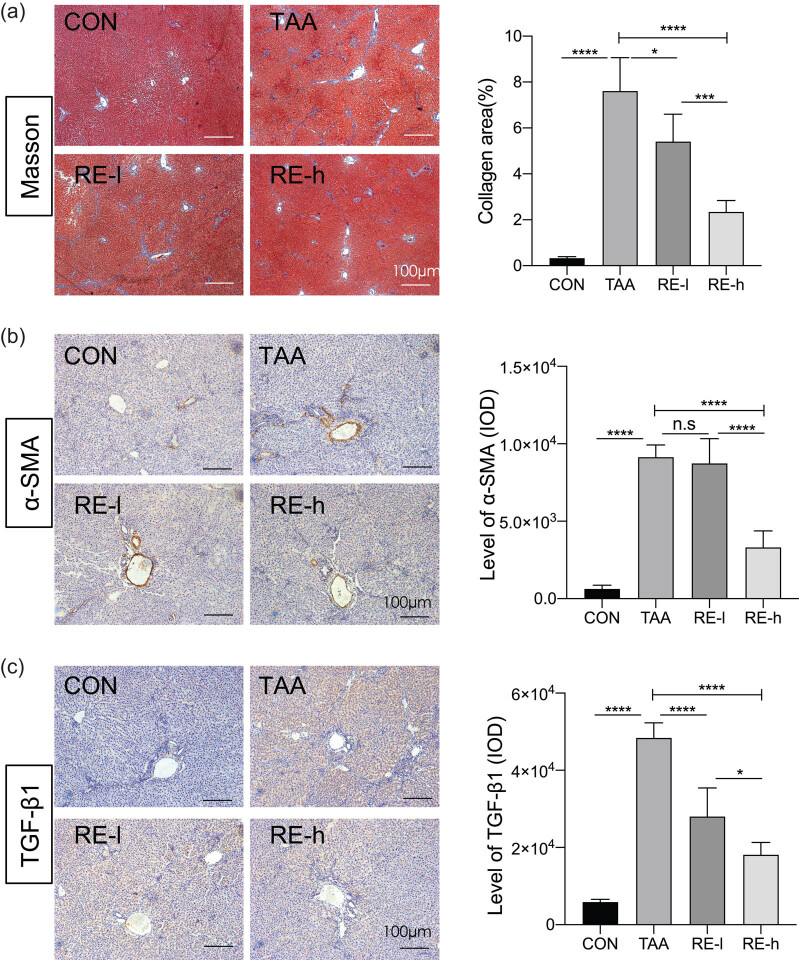
Reversine attenuated TAA-induced liver fibrosis. (a) Collagen fiber deposition was detected with Masson staining in groups, and the collagen areas of the liver in groups are listed in the bar graph. (b) α-SMA protein deposition was analyzed with immunostaining in groups, and the IOD values of α-SMA are listed. (c) TGF-β protein deposition was tested with immunostaining in groups, and the IOD values of TGF-β are listed. Data are presented as mean ± SD (*n* = 5) and analyzed with the one-way ANOVA test, with *p < 0.05, **p < 0.01, ***p < 0.001, ****p < 0.0001, n.s: no significance. Bar = 100 µm.

In the CON group, the hepatic lobule structure was normal, and the blood vessel wall showed a small amount of blue staining. However, in the TAA-treated group, a large number of collagen fibers were deposited in the portal area, central vein, and lobular area. The fiber spacing was interconnected, thus making the hepatic lobule structure irregular and confirming the TAA-induced liver fibrosis (Figure 2a). Reversine administration decreased the fibrosis fiber deposition induced by TAA. This finding was verified by a quantitative analysis, which also showed that the high reversine dose had a stronger collagen degradation effect than the RE-l (*p* < 0.001; Figure 2a). The liver tissues showed a low α-SMA expression in the smooth muscle of the vascular wall of the normal group, but its expression was prominent in the TAA-treated group (Figure 2b). The control group had low TGF-β1 protein expression, and the TAA-treated liver showed a large continuous brown-yellow area (Figure 2c). Quantitative analysis on positive areas showed that reversine significantly decreased the α-SMA and TGF-β1 expression in the TAA-treated liver (Figure 2b and c). These findings revealed that reversine attenuated TAA-induced liver fibrosis.

### 3.3 Reversine modulated inflammatory cytokines in the TAA-treated liver

**Figure 3:**
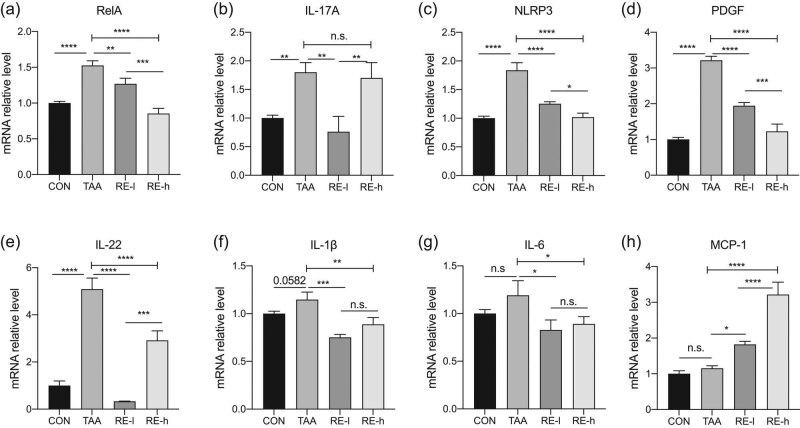
Reversine modulated inflammatory cytokines in TAA-treated liver. mRNA levels of (a) RelA, (b) IL-17A, (c) NLPR3, (d) PDGF, (e) IL-22, (f) IL-1β, (g) IL-6, and (h) MCP-1 tested by qRT-PCR in each experiment group. Values were normalized to those of ACTB and are presented as fold-changes relative to the expression level of the CON group set as 1. Data are presented as mean ± SD (*n* = 3) and analyzed with the one-way ANOVA test, with *p < 0.05, **p < 0.01, ***p < 0.001, ****p < 0.0001, n.s: no significance.

TAA increased the mRNA levels of RelA (also called NF-κb (p65) subunit, Figure 3a), IL-17A (Figure 3b), NLPR3 (Figure 3c), PDGF (Figure 3d), IL-22 (Figure 3e), and IL-1β (Figure 3f) in liver tissues, revealing the inflammatory reactions caused by TAA. Reversine administration reversed the TAA-induced increase in the inflammatory cytokine levels and the decrease in the IL-6 level (Figure 3g). In particular, the high reversine dose did not affect the IL-17A mRNA level, thereby reflecting the dose differences of reversine in modulating the inflammatory signaling. The mRNA level of MCP-1 (also called C–C motif chemokine ligand 2) was not affected by TAA but was increased by reversine in a dose-dependent manner (Figure 3h).

### 3.4 Reversine downregulated the NF-κB signaling pathway in the TAA-treated liver

**Figure 4:**
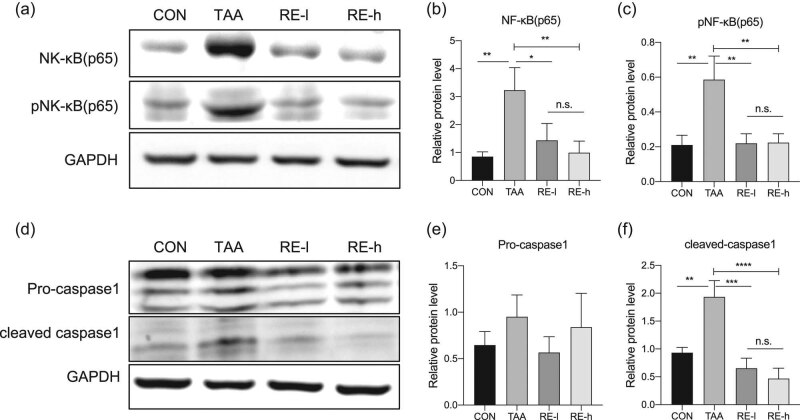
Reversine downregulated the NF-κB signaling pathway in TAA-treated liver. (a) NF-κB (p65) and phosphorylated NF-κB (p65) protein pNF-κB (p65) in each group were tested by Western blot. Relative NF-κB (p65) (b) and pNF-κB (p65) (c) levels were calculated and are listed in bar graphs. (d) Pro-caspase 1 and cleaved-caspase 1 protein in each group were tested by Western blot, and the relative pro-caspase 1 protein level (e) and relative cleaved-caspase 1 protein level (f) are listed. GAPDH was used as an internal reference. Data are presented as mean ± SD (*n* = 3) and analyzed with the one-way ANOVA test, with *p < 0.05, **p < 0.01, ***p < 0.001, ****p < 0.0001, n.s: no significance.

RelA, also known as p65, is an REL-associated protein involved in NF-κB heterodimer formation, nuclear translocation, and activation. Western blot was conducted to analyze the levels of NF-κB (p65), its phosphorylated protein (pNF-κB (p65)), and its downstream factor, namely, active caspase 1 (cleaved caspase 1). Data showed that NF-κB (p65) and pNF-κB (p65) expression levels increased significantly in the TAA-treated liver compared with those in the CON group (*p* < 0.01) but were reverted back to the normal level by the reversine treatment (Figure 4a–c). The pro-caspase 1 level showed no difference in TAA-treated livers with or without reversine treatment (*p* > 0.05; Figure 4d and e). However, reversine could significantly downregulate the TAA-induced activation of caspase 1 levels (Figure 4f). In summary, reversine alleviated the activation of the NF-κB pathway by dephosphorylating NF-κB and inactivating caspase 1.

### 3.5 XSLJZD protected TAA-injured liver without increasing fibrosis

**Figure 5:**
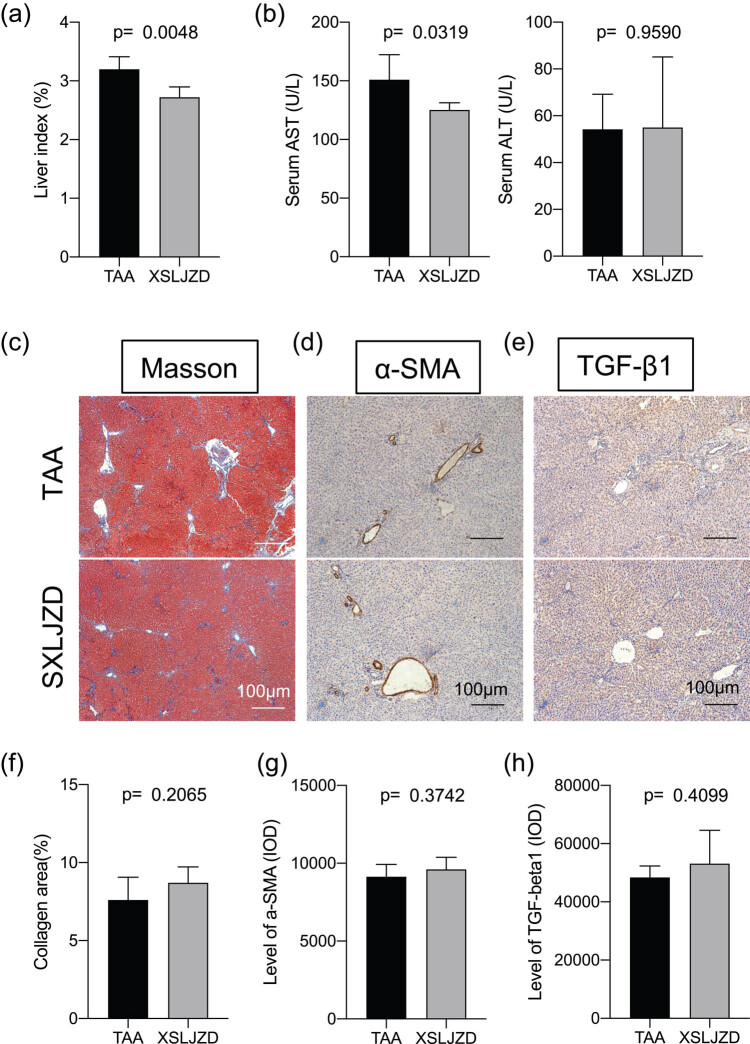
XSLJZD protected TAA-injured liver without aggravating fibrosis. TAA-injected rats treated with XSLJZD thrice per week for 3 weeks were compared with TAA-injected rats without XSLJZD administration. Liver index (a) and serum AST and ALT levels (b) are listed in bar graphs. Collagen fiber deposition detected with Masson staining (c), and α-SMA (d) and TGF-β1 (e) protein deposition detected with immunostaining are presented. Collagen area of the liver (f), and IOD values of α-SMA (g) and TGF-β1 (h). Data are presented as mean ± SD (*n* = 5) and analyzed with the unpaired *t*-test (two-tailed).

The effects of XSLJZD on TAA-induced injury were also tested. XSLJZD attenuated the liver index (Figure 5a), decreased the serum AST level (Figure 5b), and had no influence on the ALT level. Fibrosis markers collagen fiber (Figure 5c), α-SMA (Figure 5d), and TGF-β1 (Figure 5e) were compared. Quantitative analyses (Figure 5f–h) showed no difference between the two groups, indicating that XSLJZD did not aggravate the fibrosis in TAA-injured livers.

### 3.6 XSLJZD enhanced the effect of reversine on regulating inflammatory cytokines

**Figure 6:**
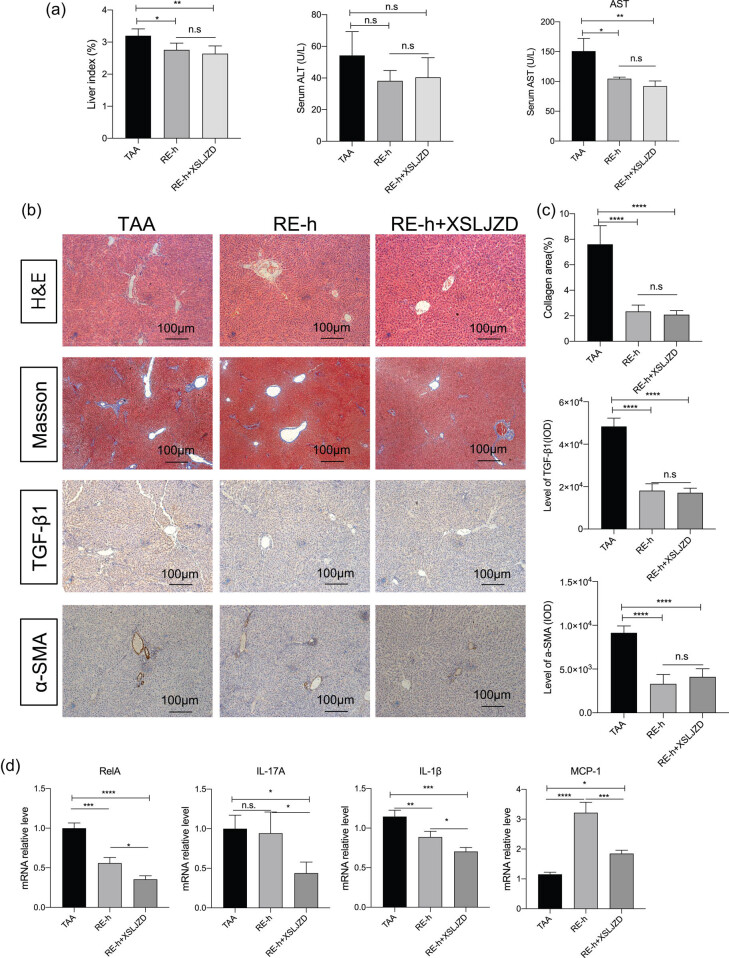
XSLJZD enhanced the effect of reversine on regulating inflammatory cytokines. The combination effects of reversine and XSLJZD were compared among the TAA group, high reversine dose group (RE-h), and RE-h + XSLJZD group. (a) Liver index, serum AST level and ALT level are listed in bar graphs. Data are presented as mean ± SD (*n* = 5) and analyzed with the one-way ANOVA test, with *p < 0.05, **p < 0.01, ***p < 0.001, ****p < 0.0001, n.s: no significance. (b) H&E staining, Masson collagen fiber staining, and TGF-β1 and α-SMA immunohistochemical staining images in each group. (c) The collagen areas of the liver, and the TGF-β1 and α-SMA protein deposition in groups are listed in the bar graph. Data are presented as mean ± SD (*n* = 5) and analyzed with the one-way ANOVA test, with ****p < 0.0001, n.s: no significance. (d) mRNA levels of RelA, IL-17A, IL-1β, and MCP-1 tested by qRT-PCR in each experiment group. Values were normalized to those of ACTB, presented as mean ± SD (*n* = 3), and analyzed the with one-way ANOVA test, with *p < 0.05, **p < 0.01, ***p < 0.001, ****p < 0.0001, n.s: no significance.

The combined effects of reversine and XSLJZD on TAA-treated rats were also analyzed. A high dose of reversine (RE-h) and co-treatment with XSLJZD (RE-h + XSLJZD) were tested and compared with TAA-treated rats with neither reversine nor XSLJZD treatment. In comparison with the TAA group, reversine combined with XSLJZD decreased the liver index (*p* < 0.01) and serum AST (*p* < 0.01). These levels were comparable to that in the RE-h group (*p* > 0.05) (Figure 6a). Histologically, reversine combined with XSLJZD improved the TAA-induced disorders of hepatic lobular structures and liver fibrosis tested by Masson collagen fiber staining and TGF-β1 and α-SMA staining (Figures 6b and c). The decreased collagen fibers and levels of TGF-β1 and α-SMA expression were comparable to that in the RE-h group (*p* > 0.05). Genetically, reversine combined with XSLJZD enhanced the inhibition effect of reversine on RelA, IL-17A, and IL-22 mRNA levels (Figure 6d). Moreover, XSLJZD reversed the increase in the MCP-1 level caused by reversine (Figure 6d), indicating their synergic effect on modulating these inflammatory cytokines.

## 4 Discussion

Hepatic fibrosis represents an important health problem worldwide with no acceptable therapy [25]. Remarkable advances have been made in the understanding of the molecular mechanisms of the development or reversal of liver fibrosis [26,27,28,29,30,31,32]. Ideal anti-fibrotic drugs do not necessarily aim to completely eliminate fibrosis but rather to attenuate its development to ensure that patients with chronic liver disease do not succumb to end organ failure. However, no therapy has satisfied these goals.

The currently available anti-fibrotic therapies mainly target HSCs and fibrogenic mediators. The points of therapeutic intervention may include efforts to remove the injurious stimuli, suppress hepatic inflammation, downregulate stellate cell activation, and promote matrix degradation [33]. Inflammatory mediators may stimulate HSC activation in chronic liver diseases. Thus, anti-inflammatory medications might be beneficial in preventing fibrosis. Potential drugs suppressing hepatic inflammation may include corticosteroids, caffeine [34,35,36], colchicine [37], and ursodeoxycholic acid [38]. Suppression or reversal of stellate cell activation is believed to be a therapeutic strategy because of the central role of HSCs in fibrogenesis [39]. The potentially available medicines may include interferon γ, rimonabant [40,41], resveratrol, quercetin, *N*-acetylcysteine [42], silibinin [43], sorafenib [44,45], and imatinib [46,47].

Low molecular weight/small molecule compounds are currently being developed to treat hepatic fibrosis via blocking cytokine receptors or intracellular signaling. One of these molecules is Y27432, a selective inhibitor of Rho-mediated focal adhesions, which prevents dimethylnitrosamine-induced hepatic fibrosis in rats [48]. Antisense molecules [49] and monoclonal antibodies [50] to the PDGF-β chain can also block experimental hepatic fibrosis. Diminazene aceturate, a small molecule used to treat human trypanosomiasis, also has anti-fibrotic properties by promoting the activity of angiotensin converting enzyme 2 in mice [51]. Our previous study showed that the aurora kinase inhibitor, reversine, inhibits the proliferation of HSCs, promotes their apoptosis [9], and inhibits the expression of TGF-β1 and α-SMA [9].

In Asian countries, herbal medicines have been used for centuries to treat liver diseases. Some studies have elucidated the cellular mechanisms of several herbal medicines with putative activity against liver fibrosis. Xiao–Chaihu–Tang, one of the most prominent herbal medicines, inhibits HSC activation and reduces fibrosis *in vitro* and *in vivo* by preventing hepatic type-I and type-III collagen expression and hydroxyproline content [52,53]. Another herbal medicine called dan-shen (*Salvia miltiorrhiza*) also inhibits fibrosis in animal models and downregulates TGF-β1, pro-collagen type-I, and type-III mRNA expression [54,55]. These studies underscore the potential value of traditional medicine, a system that has been used for centuries in many parts of the world.

Traditional therapies could lead to innovative strategies for treating hepatic fibrosis and cirrhosis. Zhao et al. [56] showed that emodin, a major active component of *Rheum palmatum* L. and *Polygonum cuspidatum*, alleviates the degree of liver fibrosis by reducing the infiltration of Gr1hi monocytes. The present study found that XSLJZD, an effective regulator of digestion in spleen Qi-deficient cases with predominant damp-cold stagnation affecting the middle jiao (middle burner), protects TAA-injured liver without aggravating fibrosis and enhances the effect of reversine on regulating RelA, IL-17A, IL-1β and MCP-1 cytokines.

In this study, reversine can reduce the increased levels of cytokines such as RelA, NLRP3, PDGF, IL-22, and IL-6 back to normal values and also increase the level of MCP-1, a profibrogenic cytokine responsible for the prolonged activation of HSCs in a paracrine or autocrine manner [57]. This side effect can be attenuated by XSLJZD co-treatment with reversine. The herbal medicine could also be used to neutralize the side effects caused by Western medicine. IL-17A, a promising therapeutic target in liver diseases [12,58], was inhibited by reversine at a low dose but was not affected by this drug at a high dose. This finding revealed that reversine exhibits dose differences in regulating IL-17A in TAA-treated liver. Given that Chinese or Asian people have a long history of TCM consumption with good tolerance to Chinese herbs, our results may serve as a guide for treating patients suffering from side effects of Western medicine by using TCMs as a complementary or alternative remedy [59,60]. The hepatoprotective effect of XSLJZD via anti-inflammatory activities improved our understanding of TCMs [20,61,62].

Chinese herbal medicine has caused liver injury in 18.6% of cases of drug-induced liver injury in China [63,64], which is a critical issue that cannot be ignored. In a randomized, double-blind, placebo-controlled trial evaluating the therapeutic effect of XSLJZT on irritable bowel syndrome (IBS), XSLJZT was orally administered to IBS patients for 28 days [23]. The liver function, including serum AST and ALT, was similar for the control and treatment groups, indicating that the oral administration of XSLJZT did not affect the liver function of patients with IBS [23]. In our present report, XSLJZD protected TAA-injured liver without aggravating fibrosis. This TCM may also enhance the effect of reversine on regulating inflammatory cytokines. All these studies indicate the efficiency of XSLJZD.

The formula XSLJZD in our study consists of nine traditional herbal substances. Although the number of compounds in a single medicinal material has been identified, identifying the specific compounds in a formula is difficult [65] because the relative content of each herb in the formula is decreased, and many trace components with low response are not easy to detect using multi-stage mass spectrometric data [65]. High-performance liquid chromatography (HPLC) coupled with linear ion trap-Orbitrap MS is an effective and reliable method to comprehensively identify the components of TCMs [65]. Shin et al. [23] analyzed the elements of XSLJZD using HPLC and found ginsenoside Rb1, ginsenoside Rg1, ginsenoside Re, costunolide, dehydrocostus lactone, glycyrrhizin, liquiritin, hesperidin, 6-gingerol, 6-shogaol, and quercetin.

The compounds in XSLJZD have different contributions. Ginsenoside Rf, one of the active ingredients of *Radix Ginseng*, can regulate intestinal motility by modulating the pacemaker activity of Cajal interstitial cells [66]. Ginsenoside Rb1 plays an anti-inflammatory role in chronic inflammatory diseases by inhibiting pro-inflammatory cytokine generation and modulating the activity of NF-κB inflammatory signaling pathways [67]. In rats, the polyacetylenic compounds of *Rhizoma Atractylodis macrocephalae* improve delayed gastric emptying [68], and *Poria* activates gastric vagal nerve activity [69]. *Pericarpium Citri reticulatae* could regulate qi and also treat indigestion [70,71]. *Rhizoma Pinelliae praeparatum* shows dose-dependent effects on bile acid transporters in mice with hepatic injury [72]. Hesperidin, a component of *Pericarpium Citri reticulatae*, can ameliorate dextran sulfate sodium-induced ulcerative colitis in mice and reduce the amount of mucosal damage, myeloperoxidase, malondialdehyde activities, and serum IL-6 levels [73]. *Fructus Amomi* was found to exhibit gastrointestinal protective, anti-inflammatory, and anti-diarrheal activities [74]. Glycyrrhizin, a major component of *Radix Glycyrrhizae recens*, can inhibit porcine epidemic diarrhea virus infection and also reduce the pro-inflammatory response by constraining the high mobility group box-protein [75]. *Radix Aucklandiae* plays a spasmolytic role in gastrointestinal motility by impeding the muscarinic receptor, 5-hydroxytryptamine receptor, and calcium influx [76]. Costunolide and dehydrocostus, two components of *Radix Aucklandiae,* reduce the severity of intestinal mucositis including diarrhea and body weight loss in a 5-fluorouracil-treated mice model by preventing ROS generation and inflammatory response [77]. A component of *Rhizoma Zingiberis recens*, 6-gingerol, shows an anti-inflammatory role in 12-O-tetradecanoylphorbol-13-acetate cyclooxygenase-2 expression by blocking the MAPK NF-κB pathway [78]. In addition, *Rhizoma Zingiberis recens* can relieve nausea [79]. Most of these compounds in XSLJZD contributed to the regulation of the inflammatory signaling, suggesting its potential therapeutic effect on inflammatory diseases.

**Figure 7:**
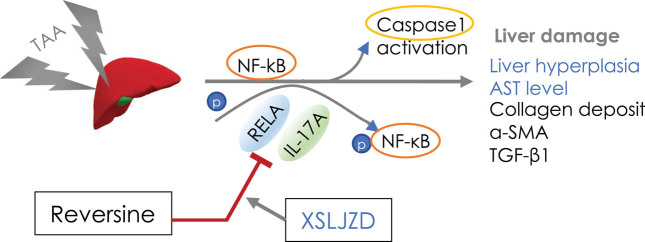
Mechanism of reversine and XSLJZD amelioration of TAA-induced hepatic injury. TAA-induced liver damage and fibrosis by activation of NF-κB/caspase-1. Reversine inhibited the TAA-induced damage by regulating the RelA/NF-κB/caspase signaling pathway. XSLJZD enhanced the suppressing effect of reversine on the inflammatory pathway.

In conclusion, our study suggested that the small chemical compound reversine has an anti-fibrotic effect on TAA-induced liver fibrosis in rats, improves hepatic function, and decreases the deposition of collagen fiber, α-SMA, and TGF-β1 in the liver. Inflammatory response modulation may be one of its underlying mechanisms. The RelA/NF-κB/caspase signaling pathway may play a pivotal role in regulating the inflammation pathway in TAA-induced liver fibrosis. Moreover, XSLJZD relieved TAA-induced increase in AST through its liver protection function, and its combination with reversine can enhance the suppressing effect on the inflammatory pathway (Figure 7). However, the specific mechanisms need further exploration.
